# Brave new RNA world(s): from prebiotic chemistry to gene regulation and RNA technology

**DOI:** 10.3389/fgene.2026.1813517

**Published:** 2026-04-15

**Authors:** Alexis A. Flores-García, Aura Hernandez-Rodriguez, E. Isabel López-Brito, Dafne F. Nava-Domínguez, Sebastian P. Pasapera, R. Franchesca Pazos-Murillo, Tonatzin Pineda-Diaz, Alberto Vázquez-Salazar

**Affiliations:** 1 Universidad Abierta y a Distancia de México, Undergraduate Program in Biotechnology Engineering, Mexico City, Mexico; 2 Departamento de Bioquímica, Centro de Investigación y de Estudios Avanzados del Instituto Politécnico Nacional, Mexico City, Mexico; 3 Escuela Nacional de Ciencias Biológicas - Instituto Politécnico Nacional, Undergraduate Program in Bacteriological and Parasitological Chemistry, Mexico City, Mexico; 4 Universidad Nacional Autónoma de México, Facultad de Estudios Superiores Zaragoza, Undergraduate Program in Biology, Mexico City, Mexico; 5 Universidad Tecnológica de México, Undergraduate Program in Pharmaceutical Chemistry and Biology, Mexico City, Mexico

**Keywords:** mRNA vaccines, noncoding RNA, prebiotic chemistry, ribozymes, RNA therapeutics, RNA world hypothesis, synthetic biology

## Abstract

RNA has long provided a plausible route by which heredity and catalysis could become linked in early evolution, and the same chemical versatility helps explain why RNA remains central to origin-of-life research, modern cell biology, and biotechnology. This review adopts a plural framing of RNA worlds to connect three regimes: a primordial RNA world constrained by geochemistry, a contemporary RNA world in which RNAs contribute to catalysis and regulation in cells, and an applied RNA world in which RNA is engineered as a programmable tool. Across these regimes, a common logic emerges from the mapping of sequence to structure to function under explicit constraints. In early evolution, cycling, interfaces, and confinement can generate heterogeneous oligomer pools and bias their persistence, whereas the transition toward Darwinian dynamics depends on copying fidelity, strand dynamics, and compartment coupled population structure. In cells and applications, noncoding RNA networks, RNA modifications, and RNA-guided targeting implement specificity in chemically complex environments, while laboratory selection and design must also confront constraints imposed by stability, delivery, and immune sensing. Across contexts, fitness landscapes and tradeoffs between peak performance and robustness provide experimental benchmarks and practical design principles for RNA function.

## 1 Introduction

The RNA world hypothesis began as a disciplined attempt to frame an origin of life problem without assuming the existence of DNA genomes and protein enzymes at the start (Crick, 1968; Orgel, 1968; Alberts, 1986; Gilbert, 1986; Lazcano, 1986). In its classical formulation, the hypothesis asks whether a single polymer could couple templated heredity to chemical function strongly enough to sustain cumulative change under selection, at least within a local window of geochemistry and time (Gilbert, 1986; Lazcano, 2012). Over the last 2 decades, that question has widened in a productive way. RNA is no longer treated only as an ancestral proxy, it is also a persistent actor in cellular chemistry and regulation, and a practical substrate for engineering in the laboratory and in medicine (Cech, 2012; Statello et al., 2021; Freije and Arechavala-Gomeza, 2025). As a result, origin-of-life research can now be read alongside contemporary RNA biology and RNA technology as three regimes that share an evolutionary logic, while differing in constraints and in what counts as a selectable phenotype (Cech, 2012; Muñoz-Velasco et al., 2024).

Following the plural framing articulated by Cech (2012), this review uses the RNA worlds concept as an organizing device rather than as stages in a single linear narrative (Cech, 2012). The primordial RNA world refers to a hypothetical regime in which RNA could, in principle, act as both genotype and phenotype, with sequence specifying a fold and a fold enabling catalysis or binding. The contemporary, or modern, RNA world refers to RNA function in modern cells, where RNA participates in catalysis and regulation through ribonucleoprotein assemblies and through networks of noncoding RNAs (ncRNAs) that shape transcription, chromatin state, RNA processing, and translation (Statello et al., 2021; Mattick et al., 2023). The RNA world of biotechnology and applications refers to engineered RNA as a programmable tool for diagnostics, therapeutics, and synthetic biology, including platforms that exploit RNA recognition, RNA catalysis, and RNA-guided nucleases (Shi et al., 2024; Bagi et al., 2025; Freije and Arechavala-Gomeza, 2025). These categories are analytical compartments defined by dominant constraints and chemistries, as well as experimentally accessible couplings between sequence and function.

The central claim developed here is that molecular evolution supplies a shared logic across all three RNA worlds, while the meaning of selection shifts with context. In the primordial RNA world, selection can begin as physicochemical filtering, for example, differential adsorption, stabilization, or compartment retention, even when copying is absent or unreliable. It can also take the form of kinetic selection during templated extension, ligation, or degradation, where variant frequencies shift due to rate differences even when heredity is imperfect. Darwinian selection, by contrast, requires heritable variation coupled to differential replication in populations, typically aided by population structure that limits parasite spread and preserves functional lineages long enough for improvement to accumulate. Throughout this review, each use of selection is tied to the relevant mechanism, and prebiotic plausibility is treated as conditional on explicit assumptions about feedstocks, concentrations, and coupling between steps, including the realistic possibility that different steps occurred in connected environments rather than within a single homogeneous setting (Ding et al., 2023; Roy, 2024; Vázquez-Salazar, 2026). This framing also provides a clean bridge to the modern applied RNA world, where selection is literal, repeatable, and instrumented, and where performance tradeoffs can be mapped rather than inferred from narrative alone (Shi et al., 2024; Freije and Arechavala-Gomeza, 2025).

## 2 The primordial RNA world

The RNA world hypothesis refers to a proposed early evolutionary phase in which RNA carried the principal informational and catalytic roles before the later expansion of protein enzymes and DNA genomes. In this sense, RNA is invoked as a polymer capable of storing sequence information, folding into defined structures, binding small molecules and metal ions, and, in some cases, catalyzing reactions, thereby linking genotype and phenotype within the same molecular class (Vázquez-Salazar and Lazcano, 2018). Contemporary formulations further emphasize that this phase need not have been chemically pure. Rather, it is best understood as RNA-centered, with salts, minerals, cofactors, and short peptides potentially shaping folding, reactivity, and persistence while RNA retained functional primacy in heredity and catalysis (Ianeselli et al., 2023; Vázquez-Salazar, 2026). Under this definition, the central question is not whether isolated RNA reactions are possible, but which boundary conditions allow activation, polymerization, copying, and retention to remain chemically continuous long enough for selection to become relevant (Muñoz-Velasco et al., 2024; Pressman et al., 2015; Robertson and Joyce, 2012).

### 2.1 Boundary conditions and chemical complexity

Within this framework, the primordial RNA world is best treated as a hypothesis space constrained by boundary conditions, not as a single canonical environment. Feedstock identities, concentrations, energy sources, and physical forcing, including cycling and gradients, determine which reaction sequences remain continuous across time rather than fragmenting into incompatible steps (Orgel, 2004; Sutherland, 2016; Walton et al., 2022; Hirakawa et al., 2025). In this review, chemical complexity denotes the combination of molecular diversity and reaction network connectivity, together with nonequilibrium physical processes that repeatedly reorganize mixtures through phase separation, adsorption, confinement, and fluctuations in water activity (Islam and Powner, 2017; Ianeselli et al., 2023). Recent syntheses emphasize that such continuity must be tested in chemically messy mixtures, where side reactions and competing sinks coexist with parallel routes and environmental filters that can enrich persistent intermediates (Vázquez-Salazar, 2026).

Complexity matters for the primordial RNA world because it influences both opportunity and clutter. Greater diversity expands the chance that useful intermediates appear, including activated nucleotides or short oligomers, but it also generates side reactions and sinks that can sequester carbon and phosphate into inert products. Physical processes such as wet-dry cycles, thermal gradients, freezing, or mineral adsorption can act as filters that concentrate subsets of products, biasing mixtures by stability, solubility, or partitioning, and thereby generating repeated opportunities for assembly and copying even when average bulk concentrations are low (Sutherland, 2016; Ianeselli et al., 2023). A key implication is that prebiotic plausibility is not a property of isolated reactions, but of coupled sequences of steps under defined physical regimes. An origin-of-life scenario must therefore specify not only a synthesis route, but also a mechanism that links synthesis, activation, polymerization, and copying without requiring mutually exclusive conditions.

### 2.2 Nucleotide and nucleoside inventories, canonical and noncanonical

A central bottleneck for the primordial RNA world remains the availability of nucleosides and nucleotides under plausible feedstocks and regimes. A major conceptual shift came from convergent routes to activated ribonucleotides that bypass the requirement to synthesize ribose and nucleobases as separable, independently optimized modules (Powner et al., 2009). This result is most useful when interpreted conditionally, it demonstrates continuity within a defined chemical regime, and it motivates a broader question that is now experimentally addressable, which coupled sets of steps remain compatible in mixtures that also contain side products and competing reactants (Islam and Powner, 2017; Walton et al., 2022).

Beyond canonical ribonucleotides, noncanonical nucleosides and nucleotide analogs may have been common. Modified bases, alternative sugar configurations, and mixed backbone chemistries could have coexisted, potentially providing both obstacles and opportunities. On one hand, heterogeneity can disrupt template copying by reducing base pairing fidelity or by altering polymerization kinetics. On the other hand, some modifications can stabilize certain folds or reduce hydrolysis, and mixed pools may widen the accessible landscape of functional structures, at least transiently. This possibility intersects with a contemporary lesson from epitranscriptomics, chemical modifications can modulate base pairing, folding, and protein recognition, suggesting that chemical diversity is not necessarily incompatible with information bearing polymers, but it complicates the mapping from genotype to phenotype (Roundtree et al., 2017; Zaccara et al., 2019). Prebiotic relevance therefore depends on whether heterogeneous pools can still support sufficient copying and selection to accumulate functional motifs.

### 2.3 Polymerization and oligomer pools, cycles, minerals, and confinement

Even if activated monomers were present, oligomer formation requires overcoming dilution, hydrolysis, and competing reactions. Nonenzymatic polymerization can be promoted by cycles that concentrate reactants, such as drying, freezing, or evaporation in porous rocks, and by mineral surfaces that align reactants or alter local water activity (Orgel, 2004; Mast et al., 2013; Ianeselli et al., 2023; Villafañe-Barajas and Vázquez-Salazar, 2026). Physical gradients can also produce non-equilibrium steady states in which polymerization outpaces degradation locally, even when the bulk environment remains unfavorable. These effects suggest a shift in emphasis from finding a single optimal chemistry to identifying regimes that repeatedly generate and retain oligomers, enabling accumulation over many cycles.

**FIGURE 1:**
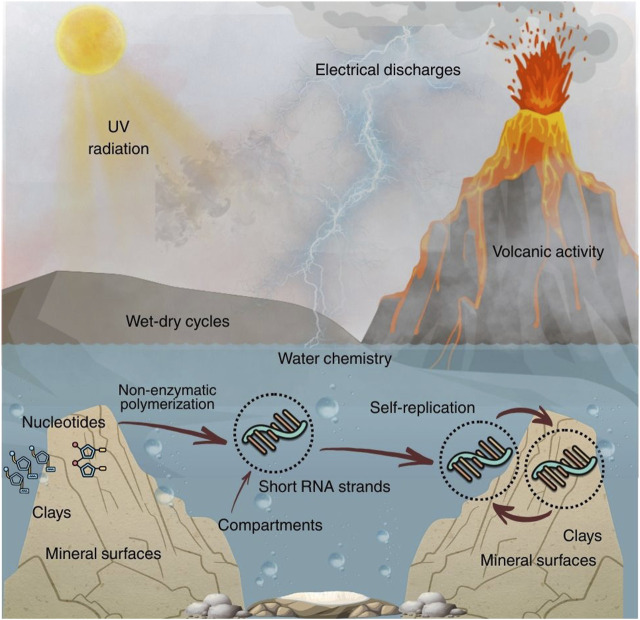
Conceptual early Earth scenario for the primordial RNA world. Energy sources such as UV radiation, electrical discharges, and volcanism, together with wet-dry cycling and aqueous chemistry, could generate and reshape organic precursors and promote the accumulation of nucleotide building blocks. Concentration on mineral surfaces and within localized microenvironments can increase effective activities and support nonenzymatic polymerization into short RNA oligomers. Compartmentalization and surface association can further stabilize oligomers and promote repeated interaction cycles, enabling some sequences to acquire catalytic activity and support limited, partial self-copying, an early bridge toward ribozyme mediated function.

Another critical point is that oligomer pools need not be uniform. Mixtures of lengths and sequences can support rudimentary network behaviors, including cooperative catalysis or templated ligation. Short oligomers can act as primers or substrates for extension, and ligation events can stitch together longer sequences that eventually cross a threshold for folding into stable secondary structures. The resulting pools may contain many nonfunctional sequences, but selection can begin to act through differential persistence, folding stability, and interaction networks, even before accurate replication is established (Walton et al., 2022; Ianeselli et al., 2023). A schematic overview of environmental energy sources and concentrating mechanisms that could connect nucleotide accumulation, nonenzymatic polymerization, compartmentalization, and partial self-copying is shown in Figure 1.

### 2.4 From differential persistence to Darwinian dynamics, three measurable replication variables

The transition from chemical evolution to Darwinian evolution can be framed in terms of measurable replication variables. One is copying fidelity, whether templated synthesis preserves sequence information across cycles strongly enough to maintain functional motifs against mutational erosion, related to the Eigen error threshold (Eigen, 1971; Higgs and Lehman, 2015). A second is strand separation and processivity, whether templates and products can cycle between paired and available states without trapping, and whether polymerization can proceed across structured segments. A third is population structure, whether compartments or spatial gradients couple genotype to phenotype by keeping functional sequences associated with their products and substrates long enough for selection to act (Joyce, 2002; Higgs and Lehman, 2015).

In this framing, the RNA world hypothesis can be tested by asking whether plausible chemistries can satisfy these variables simultaneously, not in isolation. For example, a chemistry that yields activated substrates but destroys long polymers may fail, and a chemistry that supports polymerization but traps duplexes may also fail. The modern literature is increasingly able to quantify these tradeoffs experimentally, turning broad plausibility arguments into constrained design spaces.

### 2.5 Compartments as population structure, permeability, retention, and parasite control

Compartments translate chemistry into population structure by restricting mixing and by linking local reaction effects to retention. Permeability and retention determine whether functional variants remain associated with substrates and partners, while exchange determines how rapidly parasites spread across protocell-like populations. Fatty acid vesicles provide a concrete model for this logic since nonenzymatic template-directed RNA synthesis has been demonstrated inside such compartments, coupling copying chemistry to boundary stability and permeability constraints (Adamala and Szostak, 2013). Membraneless droplets provide a complementary regime, coacervate protocells can concentrate RNA and substrates while tuning ion partitioning, yet they also introduce composition-dependent tradeoffs that can either support or inhibit RNA reactions (Drobot et al., 2018). Compartments do not guarantee Darwinian evolution, but they can increase the probability that copying, retention, and selection act on the same localized assemblies across repeated cycles (Figure 1; Higgs and Lehman, 2015).

### 2.6 RNA as genotype and phenotype, sequence to fold to function

A defining advantage of RNA in origin-of-life models is that the same polymer can encode information and implement function. Sequence determines base pairing and secondary structure, in turn secondary structure can scaffold tertiary interactions that generate binding pockets or catalytic active sites. This coupling is the heart of the RNA world hypothesis; it allows selection to act on replicating sequences while preserving causal links to chemical phenotypes. This mapping is not smooth, functional RNAs typically occupy rugged landscapes with epistasis, where combinations of mutations can be compensatory or deleterious depending on context (Peri et al., 2022; Saha et al., 2024). Such ruggedness can hinder optimization, but it can also create neutral networks that support robustness and exploration, allowing populations to drift through sequence space without loss of function until beneficial innovations become accessible. RNA function therefore arises from folding landscapes rather than from a single predetermined conformation. Secondary structure elements usually form first and constrain which tertiary contacts can later stabilize catalytically or recognition-competent states, whereas alternative helices and long-range interactions can trap the same sequence in metastable or nonproductive conformations. Sequence variation can thus alter function not only by changing local pairing but also by redistributing an ensemble across competing folds, altering the accessibility of active states, or reshaping the kinetics of strand exchange and tertiary docking. These features are now experimentally tractable. Chemical probing methods such as selective 2′-hydroxyl acylation followed by primer extension (SHAPE), increasingly coupled to high-throughput sequencing, can report on backbone flexibility and base pairing across large RNA sets, whereas nuclear magnetic resonance (NMR), cryo-electron microscopy (cryo-EM), and single-molecule fluorescence can connect conformational transitions to catalytic or regulatory output. Together, these approaches make the sequence-to-structure-to-function mapping a measurable problem rather than a descriptive metaphor (Bushhouse et al., 2022; Spitale and Incarnato, 2023).

The same rugged landscapes that shape ribozymes in origin-of-life models also shape regulatory RNAs in cells and engineered RNAs in biotechnology. In all cases, the central challenge is to understand and exploit the mapping from sequence to structure to function under constraints that reflect the environment where selection acts.

### 2.7 Ribozymes as a bridge to later systems

Ribozymes provide a bridge between the primordial RNA world and modern biology since they establish that folded RNAs can generate catalytic active sites from a limited chemical alphabet. The discovery of self-splicing introns and the catalytic RNA component of RNase P demonstrated that RNA can catalyze phosphoryl transfer chemistry without protein enzymes, converting an abstract possibility into a mechanistic fact (Kruger et al., 1982; Guerrier-Takada et al., 1983). Most known ribozymes catalyze phosphoryl transfer, yet RNA can support broader mechanistic strategies, including metal-assisted catalysis and acid-base chemistry, with strong dependence on ion composition and tertiary structure stabilization (Doherty and Doudna, 2000; Wilson and Lilley, 2009). For example, the methyltransferase ribozyme MTR1 catalyzes site-specific methyl transfer to RNA using O^6^-methylguanine as a cofactor, and structural analysis shows that a compact RNA active site can organize cofactor binding, substrate positioning, and acid-base chemistry for this reaction (Scheitl et al., 2020; Scheitl et al., 2022). Related systems extend this principle to other alkyl-transfer reactions. The SAM analogue-utilizing ribozyme (SAMURI) ribozyme uses S-adenosylmethionine or a stabilized analogue to transfer methyl or propargyl groups at defined RNA sites, demonstrating that cofactor-assisted carbon-heteroatom bond formation is experimentally accessible to RNA catalysts under selected conditions (Okuda et al., 2023; Chen et al., 2025). These examples do not imply that such chemistry was widespread in early evolution, but they materially widen the demonstrated catalytic repertoire of RNA and make discussions of catalytic breadth more concrete. For the primordial RNA world, this implies a practical criterion, catalytic feasibility is inseparable from regime definition since folding, catalysis, and backbone stability are jointly shaped by pH, ionic mixtures, and partitioning into heterogeneous microenvironments (Wilson and Lilley, 2009).

## 3 The contemporary RNA world: RNA as catalyst, guide, and regulatory logic in cells

The contemporary RNA world does not denote a return to primordial chemistry, but the persistence and expansion of RNA-centered functions within protein-dominated cells. Modern biology still depends on RNA as catalyst, structural scaffold, molecular guide, metabolite sensor, and chemically modulated information carrier. In that sense, the contemporary RNA world entails more than the survival of a few catalytic relics. It includes ribonucleoprotein machines in which RNA remains central to core reactions, as well as extensive networks of ncRNAs that route gene expression, chromatin organization, RNA processing, translation, genome defense, and cell-to-cell communication through sequence complementarity and structure-dependent recognition (Mattick et al., 2023; Muñoz-Velasco et al., 2024).

### 3.1 Catalytic RNAs and RNA at the core of translation

The most direct evidence for an active contemporary RNA world lies in catalytic RNAs embedded in essential cellular machines. The ribosome remains the clearest example, peptide bond formation occurs in an active site built primarily from ribosomal RNA (rRNA), showing that RNA catalysis persists at the center of translation rather than only in hypothetical early stages of evolution (Nissen et al., 2000). RNase P provides a conceptually distinct case, in which an RNA subunit supplies the catalytic activity required for endonucleolytic processing of precursor-transfer RNAs (pre-tRNAs), whereas protein contributions vary across lineages and mainly modulate substrate recognition and stability (Guerrier-Takada et al., 1983). A third example comes from the spliceosome. Small nuclear RNAs (snRNAs) assemble with proteins into small nuclear ribonucleoproteins, where mechanistic as well as structural analyses support RNA-mediated chemistry at the catalytic center of pre-messenger RNA (pre-mRNA) splicing, reinforcing that RNA can directly participate in phosphoryl transfer even within large multicomponent ribonucleoprotein assemblies (Fica et al., 2013; Karijolich and Yu, 2010). Taken together, these systems support a conservative but important conclusion, RNA catalysis in modern cells is not merely vestigial. It remains embedded in processes where RNA provides catalytic architecture, substrate positioning, or reaction specificity, while proteins contribute stabilization, remodeling, and auxiliary chemistry.

### 3.2 Regulatory RNAs, noncoding layers of genetic control

**FIGURE 2:**
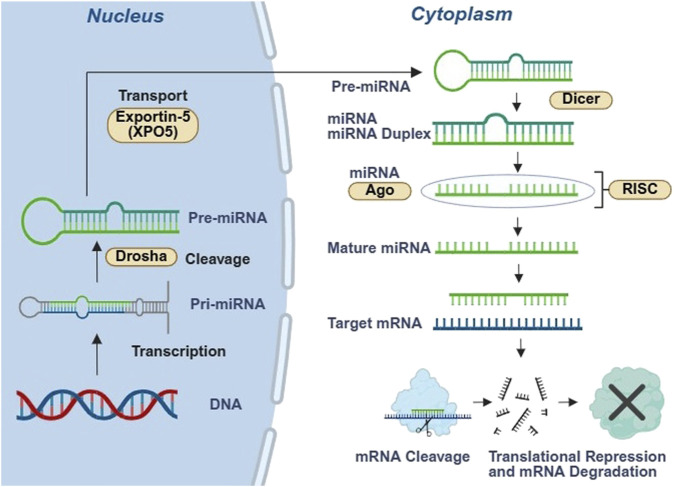
MicroRNA biogenesis and RNA-guided post-transcriptional regulation in the contemporary RNA world. Primary microRNA transcripts (pri-miRNA) are transcribed in the nucleus and processed by Drosha into precursor microRNAs (pre-miRNA), which are exported to the cytoplasm by Exportin-5 (XPO5). Dicer cleavage generates a miRNA duplex that is loaded into Argonaute (Ago) to form the RNA-induced silencing complex (RISC). Strand selection yields a mature miRNA guide that directs RISC to complementary sites in target mRNAs, resulting in mRNA cleavage or translational repression coupled to mRNA decay, depending on pairing and cellular context.

A defining feature of the contemporary RNA world is that RNA functions as a programmable recognition layer that routes regulatory processes through complementarity, folding, and selective binding. MicroRNAs (miRNAs) are short RNAs that guide Argonaute proteins to partially complementary sites in target transcripts, thereby modulating translation and transcript stability through sequence-directed recognition and recruitment of effector complexes (Figure 2; Bartel, 2018). Their output depends not only on target complementarity, but also on regulated biogenesis and kinetic competition during processing and loading, which makes microRNA control sensitive to developmental state, cell type, and stress context (Treiber et al., 2019). Small interfering RNAs (siRNAs) provide a related but mechanistically distinct mode of regulation, typically using near-complete complementarity to direct transcript cleavage or repression. Together with microRNAs they illustrate how short RNAs can implement programmable post-transcriptional control at scale (Lam et al., 2015).

Long noncoding RNAs (lncRNAs) broaden this logic from transcript targeting to nuclear organization. Rather than operating through a single canonical mechanism, lncRNAs can act through localization-dependent interactions with chromatin regulators, transcriptional machinery, RNA-binding proteins, and other RNAs. Their functional interpretation therefore depends on expression level, subcellular distribution, and perturbation strategy, not simply on sequence conservation alone (Statello et al., 2021; Mattick et al., 2023). Circular RNAs (circRNAs) extend this landscape further. Their covalently closed topology often increases stability, and many can function as protein-binding platforms, regulators of RNA availability, or, in selected cases, templates for translation, although broad claims require stringent validation at endogenous abundance and with appropriate genetic controls (Kristensen et al., 2019).

Other ncRNA classes show that this modern RNA world is broader than the now standard trio of miRNAs, lncRNAs, and circRNAs. Piwi-interacting RNAs associate with Piwi proteins and play central roles in germline genome defense by suppressing transposable elements, thereby coupling RNA-guided recognition to maintenance of genome integrity across generations (Tóth et al., 2016). Bacterial and archaeal systems add further support to the same general principle. Riboswitches are structured noncoding RNAs that sense metabolites or cofactors directly and alter transcriptional or translational outputs without requiring protein factors, which makes them particularly relevant to an RNA world framing because they unite ligand recognition with regulatory control in a single RNA architecture (Kavita and Breaker, 2023). Additional specialized classes, including enhancer RNAs and extracellular RNAs, extend RNA-mediated regulation to local transcriptional activation and intercellular signaling, respectively, showing that RNA function in modern cells is distributed across multiple scales of biological organization (Arnold et al., 2020; Wu et al., 2022). The resulting picture is not one of isolated RNA curiosities, but of a layered regulatory economy in which RNA repeatedly supplies specificity, adaptability, and network integration.

### 3.3 Epitranscriptomics and RNA as a chemically modulated information layer

The contemporary RNA world also includes a chemical dimension. RNA is not a fixed alphabet written once and read passively. It is a chemically modulated information layer in which nucleotide modifications alter folding, decoding, recognition, localization, and decay. This is evident in transfer RNAs (tRNAs) and rRNAs, where modifications influence translational accuracy, structural stability, and ribosome performance, and it extends to messenger RNAs (mRNAs) and regulatory transcripts, where transcriptome-wide mapping has broadened the scope of epitranscriptomics as a regulated layer of RNA control (Roundtree et al., 2017; Zaccara et al., 2019).

A particularly important point, often underemphasized in broad discussions of RNA regulation, is that many of these modifications are themselves directed by ncRNAs. Small nucleolar RNAs (snoRNAs) guide 2′O-methylation and pseudouridylation of rRNAs, modifications that are central to ribosome assembly and function, while related small Cajal RNAs (scaRNA) direct analogous modifications in spliceosomal small nuclear RNAs (snRNAs), thereby tuning the molecules that carry out splicing (Huang et al., 2022; Darzacq et al., 2002). In this sense, the contemporary RNA world is recursive, RNA molecules do not merely encode or regulate information, they also help transfer the chemical marks that determine how other RNAs will fold and function.

This chemically modulated view of RNA also intersects with innate immune recognition. Certain nucleoside modifications can reduce the recognition of RNA by Toll-like receptors and related sensors, which is relevant both for cellular discrimination between self and nonself and for the design logic of therapeutic RNAs, where modified nucleosides can reduce inflammatory signaling while preserving translational output (Karikó et al., 2005). This emphasizes that RNA behavior in cells is inseparable from chemical context. The contemporary RNA world therefore entails catalytic RNAs, guide RNAs, regulatory RNAs, and chemically modified RNAs acting together in an integrated system where sequence, structure, modification state, localization, and protein partners jointly determine function.

## 4 The modern applied RNA world: RNA as a programmable material

### 4.1 Engineering RNA substrates, from selection to design

Applied RNA technology draws on the same genotype to phenotype mapping as origin-of-life models and cellular RNA biology, but it operates with explicit selection and design goals. *In vitro* selection, or SELEX, established that functional nucleic acids can be isolated directly from random libraries by iterative enrichment for binding or catalysis, thereby converting sequence space into a searchable design substrate (Ellington and Szostak, 1990; Robertson and Joyce, 1990; Tuerk and Gold, 1990). Modern implementations combine library design, high-throughput sequencing, and quantitative readouts to map functional landscapes and identify sequence features that govern performance and robustness (Saha et al., 2024). This approach is central to aptamer development, ribozyme engineering, and RNA device construction.

### 4.2 RNA therapeutics, constraints from delivery, stability, and immune sensing

RNA therapeutics span antisense oligonucleotides, siRNAs, mRNA delivery platforms, and guide RNAs used for genome editing. Across modalities, the main constraints are not only target recognition, but also stability in biological fluids, delivery into the right cells and compartments, and avoidance of inappropriate innate immune activation (Paunovska et al., 2022; Freije and Arechavala-Gomeza, 2025). Chemical modifications can reduce immune sensing and increase stability, but they can also alter folding and interaction networks, requiring careful balancing of activity and robustness (Karikó et al., 2005; Paunovska et al., 2022). These constraints are conceptually similar to origin-of-life constraints, context defines which sequences are functional, and selection for peak activity in a simple assay may fail during transfer into complex environments.

### 4.3 Case study, mRNA vaccines and the rapid scaling of RNA medicine

The rapid deployment of mRNA vaccines against SARS-CoV-2 offers a clear case study in how RNA performance is set by coupled constraints rather than by sequence alone. The core problem was to deliver an RNA that is both stable enough to persist through handling and extracellular exposure, and readable enough to support high protein expression after cytosolic release, while avoiding premature clearance or excessive innate immune activation. Solutions were implemented at multiple layers, including mRNA architecture, purification, and delivery. Transcript design incorporated a 5′ cap, optimized untranslated regions, a tuned poly(A) tail, and codon choices that improve translation and reduce deleterious RNA structures. At the chemistry layer, incorporation of modified nucleosides, together with stringent control of double-stranded RNA byproducts from *in vitro* transcription, reduced activation of RNA sensing pathways while preserving translational output (Karikó et al., 2005; Pardi et al., 2018). At the delivery layer, lipid nanoparticles provided protection from nucleases, promoted cellular uptake, and enabled endosomal escape through ionizable lipid behavior that depends on pH and membrane composition, making formulation chemistry inseparable from biological context (Pardi et al., 2018; Paunovska et al., 2022).

What made this episode distinctive was not only technical feasibility, but also the speed at which optimization, manufacturing, and clinical validation converged. Large-scale *in vitro* transcription and chromatographic purification pipelines were aligned with formulation processes that reproducibly control particle size, encapsulation efficiency, and batch consistency, parameters that directly affect dose, reactogenicity, and expression kinetics *in vivo* (Paunovska et al., 2022). Phase 3 trials then provided the clinical proof that these coupled design choices translated into strong protection against COVID-19 (Polack et al., 2020; Baden et al., 2021). In parallel, real-world deployment exposed the remaining context constraints that continue to shape the platform, including cold chain requirements, stability during storage, and the need to balance innate immune stimulation that can support adjuvanticity against inflammation that can limit tolerability. The broader takeaway for the applied RNA world is that RNA medicines scale when they are engineered as integrated systems: sequence, chemical modification, impurities, formulation, and delivery route jointly define function, and robustness across variable physiological environments is treated as a primary design objective rather than an afterthought (Pardi et al., 2018; Paunovska et al., 2022).

### 4.4 RNA devices, diagnostics, and CRISPR workflows

RNA-based diagnostics and synthetic biology devices often couple programmable recognition to an amplifiable output. CRISPR-based detection platforms exploit collateral nuclease activities, for example, Cas13-triggered RNA cleavage and Cas12-triggered single-stranded DNA cleavage, to generate signal amplification after target recognition, providing a compact molecular architecture that separates specificity, encoded in a guide RNA, from reporting chemistry (Gootenberg et al., 2018; Chen et al., 2018; Bagi et al., 2025). RNA switches and riboswitch-inspired designs provide a complementary strategy, ligand binding or conditional folding is used to gate cleavage, translation, or assembly, effectively turning RNA structure into a control element rather than a passive scaffold (Serganov and Patel, 2007; Doherty and Doudna, 2000; Mustafina et al., 2021). A recurring theme across these applications is context dependence, RNA folding and activity depend on ion composition, crowding, competing nucleic acids, and folding history, which motivates selection and validation regimes that approximate deployment conditions rather than relying exclusively on idealized buffers (Doherty and Doudna, 2000; Bagi et al., 2025).

### 4.5 Ribozymes and aptamers as modular RNA tools, switching, targeting, and compartmentalized deployment

Ribozymes and aptamers are compact, genetically encodable RNAs that translate sequence into function through predictable base pairing and fold-dependent recognition. Ribozymes implement catalysis, commonly RNA cleavage, ligation, or template extension, whereas aptamers are structured RNAs selected for high affinity binding to a target ligand, protein, or nucleic acid. A central enabling concept is that function can be discovered and optimized directly from sequence libraries via iterative selection and amplification workflows, which originally established aptamer selection *in vitro* and were later generalized into modern high-throughput pipelines for RNA discovery (Ellington and Szostak, 1990; Robertson and Joyce, 1990; Tuerk and Gold, 1990; Yokobayashi, 2020).

**FIGURE 3:**
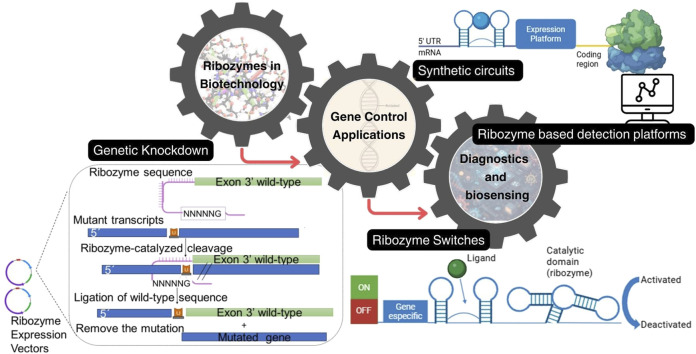
Ribozyme-based architectures for gene control, synthetic regulation, and diagnostics in the RNA world of biotechnology. Catalytic RNAs convert sequence-encoded folding into functional outputs that support modular applications. Left, ribozyme-mediated gene-control via targeted RNA cleavage for knockdown, and splice-based transcript repair concepts in which a designed trans-splicing RNA module redirects RNA processing to replace a mutated segment with a desired sequence. Bottom, aptazyme or ribozyme-switch designs couple a ligand-binding aptamer sensor to a catalytic ribozyme domain, converting ligand recognition into ON or OFF regulation through fold-dependent activation or deactivation. Top, synthetic regulatory elements placed in the 5′ UTR gate translation by altering RNA structure and ribosome access, enabling programmable expression platforms that can be assembled into larger synthetic circuits. Right, these RNA modules can be integrated into ribozyme-based detection platforms for diagnostics and biosensing, where biochemical recognition is converted into a measurable signal for monitoring and analysis.

In gene control applications, self-cleaving ribozymes and aptamer-ribozyme engineered constructs (aptazymes) provide a direct route to post-transcriptional regulation as cleavage changes RNA stability, translation, or localization, and therefore creates a tunable layer of control downstream of transcription. In practice, reliable switching depends less on the chemical step of cleavage and more on folding and kinetic partitioning inside long transcripts that contain competing structures and context-dependent transcriptional dynamics. Synthetic scaffolds for mammalian riboswitch-like control illustrate how engineered architectures can be made modular while still requiring empirical mapping of sequence to function in the intended cellular context (Figure 3; Serganov and Patel, 2007; Mustafina et al., 2021; Yokobayashi, 2020).

A second applied lever is physical format. Compartmentalization can stabilize components, tune-exchange with the environment, and improve robustness in the presence of nucleases or inhibitors. Encapsulation of cell-free biosensors in lipid vesicles exemplifies how membrane permeability and compartment composition can become design parameters that feedback on kinetics and detection thresholds (Boyd et al., 2023).

Finally, therapeutic relevance depends on integrated constraints. For aptamers and catalytic RNAs, *in vivo* performance is shaped by nuclease stability, off-target recognition, immune sensing, and delivery, so potency in a clean buffer is rarely the dominant determinant of clinical utility. As a result, this deployability is treated as a coupled property of folding, pharmacokinetics, and tissue access, often supported by chemical modification and formulation strategies (Zhou and Rossi, 2017; Paunovska et al., 2022; Freije and Arechavala-Gomeza, 2025). Representative ribozyme-based architectures for gene control, ligand responsive switching, synthetic circuit design, and diagnostic biosensing are summarized in Figure 3.

## 5 Bridging principles across RNA worlds, evolution-guided design rules

### 5.1 A shared abstraction, fitness landscapes under constraints

Fitness landscapes offer a common language for relating sequence variation to functional performance across the three RNA worlds, even though the observable used as fitness differs by context (Saha et al., 2024). In the primordial RNA world, fitness proxies are often physicochemical and kinetic, for example, differential persistence against degradation, copying and ligation rates, and retention within compartments or at interfaces, all of which shape enrichment over repeated environmental cycles (Saha et al., 2024). In the contemporary RNA world, fitness proxies shift toward regulatory and informational performance, including interaction specificity, regulatory impact, and compatibility with cellular RNA processing and surveillance (Statello et al., 2021). In the RNA world of biotechnology and applications, fitness becomes explicitly operational and deployment-focused, with proxies such as potency relative to off-target effects, delivery efficiency, manufacturability, and stability during storage and *in vivo* exposure to nucleases and immune sensing (Paunovska et al., 2022). For catalytic and binding RNAs in particular, operational fitness often integrates catalytic rate or binding kinetics with performance in complex matrices and with tolerance to design features introduced for stability, switching, or delivery (Yokobayashi, 2020; Fukunaga et al., 2023; Zhou and Rossi, 2017).

A practical implication is methodological, fitness landscapes should be mapped under conditions that approximate the intended environment of action, rather than only under a single optimized buffer. Catalytic RNA landscapes can change measurably when environmental variables are perturbed, indicating that relative sequence ranking, epistasis, and accessible trajectories can be condition-dependent (Peri et al., 2022). In applied settings, the same principle motivates selection and screening designs that explicitly incorporate constraints that later determine utility, including ionic composition, crowding, and degradation pressures (Saha et al., 2024; Paunovska et al., 2022).

### 5.2 Robustness versus efficiency trade-offs

Across RNA worlds, peak activity and robustness often pull in different directions. High affinity binding and efficient catalysis frequently rely on tightly tuned tertiary structure, which can increase sensitivity to mutation and to physicochemical shifts that reshape folding ensembles, for example, changes in Mg^2+^ activity, temperature, or solvent conditions (Saha et al., 2024). By contrast, sequences embedded in broader neutral networks can tolerate perturbations with smaller performance losses, although they may not reach the same maxima under narrowly optimized conditions (Saha et al., 2024). This tradeoff is relevant to the primordial RNA world, where fluctuating regimes can favor motifs that remain recoverable and recurrent across cycles, even if they are not optimal in any single phase. It is equally relevant to the RNA world of biotechnology and applications, where selection and engineering benefit from rewarding performance across a defined range of conditions that mimic storage, delivery, and intracellular environments, rather than privileging a single assay condition (Paunovska et al., 2022; Peri et al., 2022).

### 5.3 Modularity, recombination, and chimeric strategies

Modularity is a recurrent route to novelty when *de novo* invention is constrained by chemistry, kinetics, or design complexity. In the primordial RNA world, ligation and recombination among short fragments can generate new architectures without requiring long processive copying, and experimental models show that interacting RNA replicators can form cooperative networks whose persistence depends on mixing and population structure (Kakizaki and Mizuuchi, 2025; Mizuuchi et al., 2022; Vaidya et al., 2012). In the contemporary RNA world, short guide RNAs redirect conserved effector cores, exemplified by CRISPR systems where guide sequence changes retarget a largely invariant nuclease machinery (Jinek et al., 2012). In the RNA world of biotechnology and applications, similar modular logic supports rapid prototyping, including programmable RNA regulators and chimeric constructs that combine sensing and actuation through designed base pairing rules (Green et al., 2014).

Modularity also introduces predictable failure modes. Combining domains increases the number of alternative base pairing options, which can generate off-pathway folds and reduce activity or specificity. For this reason, modular design increasingly relies on approaches that expose hidden epistasis and misfolding routes, including deep mutational scanning, high-throughput activity measurements, and iterative selection cycles that penalize undesired conformations under relevant conditions (Saha et al., 2024; Fowler and Fields, 2014). In practice, this is where mechanistic ribozyme knowledge and landscape mapping meet, since structural motifs that enable catalysis can also generate kinetic traps that only become visible when sequence space is scanned at scale (Müller et al., 2016; Saha et al., 2024).

### 5.4 Outlook, RNA nanotechnology and cell-free systems

Two methodological developments are bringing the three RNA worlds into tighter dialogue. RNA nanotechnology provides ways to impose geometry on RNA function by arranging ribozymes, aptamers, and guide RNAs on designed scaffolds, thereby tuning effective molarity, coupling, and cooperative behavior in a physically interpretable manner (Figure 3; Stewart, 2024). Foundational work framed this as an emerging field in its own right, with direct relevance to multi-component RNA devices where spatial organization can matter as much as further optimization of an already competent catalytic core (Guo, 2010). In parallel, cell-free systems, including defined transcription and translation extracts and reconstituted biochemical modules, provide testbeds for prototyping and selection under controlled ionic and degradation constraints, which can shorten the feedback loop between mechanistic hypotheses and measurable performance (Karim et al., 2020). When combined with compartmentalization, for example, vesicle encapsulation, cell-free platforms can also approximate exchange and protection constraints that are central both to field deployment and to protocell style population structure (Boyd et al., 2023). Together, these platforms support a practical research strategy, parameterize how fidelity, strand dynamics, and compartment coupling respond to the same environmental variables that shape RNA performance in prebiotic regimes and in biotechnology (Saha et al., 2024).

## 6 Experimental approaches that connect molecular evolution and RNA world models

### 6.1 *In vitro* evolution and selection of functional RNAs

*In vitro* selection provides a controlled way to test how RNA function can emerge from large pools of sequence variation under explicitly defined constraints. SELEX and related selection-amplification workflows enrich RNA libraries for binding or catalytic phenotypes over repeated rounds, making it possible to measure how stringency, mutation, and environmental parameters shape what is recovered from the same starting diversity (Tuerk and Gold, 1990; Ellington and Szostak, 1990; Robertson and Joyce, 2012). For RNA world-motivated questions, the main value is not only that functional RNAs exist, but that the experimental format allows direct manipulation of variables that were likely central in early settings, such as ion mixtures, temperature, hydration state, and compartmentalization, while keeping the genotype-to-phenotype link experimentally traceable (Robertson and Joyce, 2012; Saha et al., 2024).

### 6.2 What artificial selection reveals about early evolutionary trajectories

Artificial selection experiments sharpen several expectations that matter for early evolution scenarios. First, fitness landscapes are often rugged, improvement commonly depends on epistasis, meaning combinations of substitutions can be required to cross local constraints rather than accumulating purely additive gains (Saha et al., 2024). Second, neutrality is common, many variants preserve a fold or a function above a threshold, which allows populations to explore sequence space through networks of related sequences while maintaining activity (Robertson and Joyce, 2012; Saha et al., 2024). Third, selection outcomes depend strongly on what is measured and under which conditions it is measured. Selections performed under narrow laboratory optima can enrich high peak activity variants that transfer poorly once ionic strength, temperature, crowding, or competing interactions are changed. By contrast, designs selected across multiple conditions, or under periodically perturbed conditions, more often show broader operating ranges, sometimes at the cost of peak performance. This trade-off is directly relevant when using laboratory evolution to approximate early environmental variability, or when engineering RNAs expected to operate in complex biological or diagnostic matrices (Saha et al., 2024).

### 6.3 Case studies, polymerase ribozymes, ligases, and regulatory RNAs

Polymerase ribozymes provide a uniquely direct bridge between RNA world models and laboratory evolution since they couple catalytic function to the possibility of heredity by RNA-templated RNA synthesis. Demonstrations that a polymerase ribozyme can amplify RNA established an experimental foothold for studying RNA-based replication using measurable performance variables such as processivity, fidelity, and sensitivity to inhibition by products or by competing sequences (Horning and Joyce, 2016). A major recent advance is the discovery of QT45, a 45-nucleotide polymerase ribozyme selected *de novo* from random sequence pools that catalyzes general RNA-templated RNA synthesis using trinucleotide triphosphate substrates in mildly alkaline eutectic ice (Gianni et al., 2026). In that work, QT45 synthesized its complementary strand using a random triplet pool with 94.1% per nucleotide fidelity, and it synthesized a copy of itself using defined triplet substrates, with yields on the order of 0.2 percent over 72 days. Although these yields remain low, the experiments directly address a central tension in RNA world arguments, polymerase function may be encoded by much smaller motifs than previously assumed, and freezing-driven concentration regimes can support measurable copying with fidelity values that connect naturally to error threshold constraints (Eigen, 1971; Gianni et al., 2026). Structural resolution has added a complementary layer, cryo-EM has linked polymerase ribozyme architectures to a functional landscape, enabling mechanistic interpretation of constraints and guiding targeted evolutionary tests (McRae et al., 2024). Importantly, polymerase ribozymes can now be placed under sustained RNA-catalyzed evolution, making it possible to quantify adaptation while directly tracking tradeoffs between speed, accuracy, and robustness under a defined microenvironment (Papastavrou et al., 2024; Saha et al., 2024). Ligase ribozymes and self-cleaving motifs remain informative in parallel, since they support high-throughput measurement of rate and specificity, and they allow controlled tests of context dependence, including how folding pathways and mixture composition alter function. Together, these case studies make it possible to align mechanistic chemistry with evolutionary dynamics in a way that is difficult to achieve for most prebiotic steps in isolation (Saha et al., 2024).

### 6.4 High-throughput methods, deep mutational scanning, and fitness landscape mapping

A major methodological shift is the move from anecdotal winners to quantitative maps. Deep mutational scanning and related high-throughput genotype-to-phenotype approaches measure activity proxies across thousands to millions of variants, typically by linking function to a sequencing-readable output, which converts concepts such as epistasis, robustness, and neutral neighborhoods into datasets that can be compared across conditions (Fowler and Fields, 2014; Saha et al., 2024). As emphasized above, landscape structure is condition-dependent, so a key advantage is that the same sequence space can be interrogated under multiple regimes, for example, by varying Mg^2+^ availability, introducing mixed substrates, adding compartments, or changing cycling schedules, and then asking which constraints dominate and which tradeoffs recur (Peri et al., 2022; Saha et al., 2024). These landscape measurements become more informative when they are paired with direct structural readouts. Chemical probing methods such as SHAPE, NMR, cryo-EM, and single-molecule approaches can resolve tertiary contacts and conformational exchange in selected systems. This allows fitness effects to be assigned more precisely, distinguishing altered catalytic chemistry from ensemble redistribution, kinetic trapping, or misfolding (Bushhouse et al., 2022; Spitale and Incarnato, 2023; McRae et al., 2024; Papastavrou et al., 2024; Saha et al., 2024).

## 7 Discussion

**FIGURE 4:**
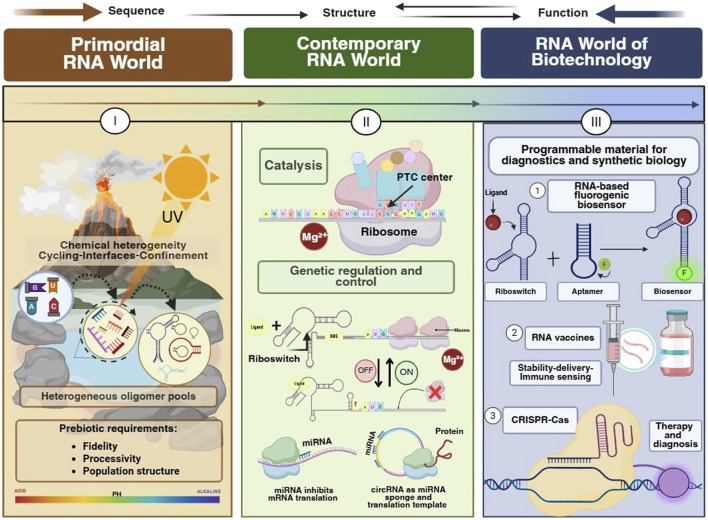
The three RNA worlds framework and a shared evolutionary logic. The scheme links three regimes through a common mapping from sequence to structure to function, while highlighting that constraints and selectable phenotypes differ by context (I) Primordial RNA world, chemical heterogeneity plus cycling, interfaces, and confinement shape heterogeneous oligomer pools and determine whether replication relevant variables such as fidelity, strand dynamics, and population structure can be satisfied. (II) In the contemporary RNA world, RNA participates in catalysis and in a programmable recognition layer that supports gene regulation and control in crowded cellular environments. (III) RNA world of biotechnology, RNA is used as a programmable material for diagnostics, therapeutics, and synthetic biology, with performance limited by stability, delivery, and immune sensing.

The RNA worlds framing is most useful when treated as a constraint map rather than as a single historical storyline (Figure 4; Cech, 2012; Muñoz-Velasco et al., 2024). Across primordial chemistry, modern genetics, and applied biotechnology, the core continuity is the mapping from sequence to structure to function, and the core discontinuity is the environment that defines what counts as a selectable phenotype (Cech, 2012; Saha et al., 2024). This distinction clarifies why the RNA world hypothesis remains scientifically productive even when individual prebiotic steps remain debated, and why modern RNA biology and RNA engineering can be used to operationalize origin-of-life questions as measurable problems rather than as narrative plausibility arguments (Pressman et al., 2015; Robertson and Joyce, 2012; Muñoz-Velasco et al., 2024; Vázquez-Salazar and Muñoz-Velasco, 2025).

### 7.1 Selection is mechanism-specific across regimes

In primordial settings, enrichment can arise through physicochemical filtering and kinetic bias before reliable heredity exists. Differential persistence, differential partitioning at interfaces, and rate differences during templated extension or ligation can shift sequence frequencies in reproducible ways without requiring full Darwinian replication (Ianeselli et al., 2023; Walton et al., 2022). This is a useful clarification because it separates two questions that are often conflated, whether prebiotic environments can generate and retain RNA-like polymers at all, and whether those polymers can enter a regime of lineage-based selection. The first question is increasingly addressed by systems chemistry and by explicit regime mapping in chemically complex mixtures (Islam and Powner, 2017; Ianeselli et al., 2023; Vázquez-Salazar, 2026). The second question depends on a smaller set of replication variables, copying fidelity, strand dynamics, and population structure via compartments or spatial gradients (Eigen, 1971; Higgs and Lehman, 2015).

In modern cells, selection is filtered through genetics and regulation. RNAs rarely act alone, they operate under strict genetic control, processing, and protein-assisted networks where sequence-specific recognition has phenotypic consequences only when embedded in coupled systems (Bartel, 2018; Statello et al., 2021; Mattick et al., 2023). This point matters for how origin-of-life-inspired logic is transferred to biology, functional output is not determined only by an RNA fold, it is determined by compatibility with cellular binding proteins, modifications, and pathway kinetics. The epitranscriptome provides a direct example, chemical modifications modulate pairing and recognition, but their biological meaning depends on writers, erasers, and readers rather than on chemistry alone (Roundtree et al., 2017; Zaccara et al., 2019).

### 7.2 Small polymerase ribozymes sharpen plausibility debates

A recurring critique of RNA world models is the apparent paradox that polymerase ribozymes must be complex enough to catalyze templated synthesis, yet large size and structural complexity impede copying and make spontaneous emergence implausible. Class I-derived polymerase ribozymes have provided powerful mechanistic footholds, including RNA amplification and structural interpretation, but they have also tended to be long and structurally elaborate (Horning and Joyce, 2016; McRae et al., 2024). The discovery of QT45 directly shifts this landscape. QT45 demonstrates that a short motif can encode polymerase activity and can support partial self-copying under defined regimes, using triplet substrates in eutectic ice and achieving fidelity values that naturally connect to error threshold constraints (Gianni et al., 2026; Eigen, 1971). Even if such systems remain far from open-ended evolution, they turn a long-standing qualitative argument into an experimentally-grounded design space, one can now ask how often small polymerase motifs occur in sequence space, which regimes stabilize their folds while permitting turnover, and which ecological structures suppress parasitism long enough for improvement to accumulate (Higgs and Lehman, 2015; Papastavrou et al., 2024).

### 7.3 Fitness landscapes unify origins, genetics, and engineering

A second unifying implication is methodological. Fitness landscapes are not abstract metaphors, they can be measured, perturbed, and compared across environments. For early evolution questions, landscape mapping provides a way to test sufficiency criteria under regime changes, for example, whether a motif that performs in one ionic mixture remains functional under cycling, confinement, or heterogeneous substrates (Peri et al., 2022; Saha et al., 2024). For genetics, landscapes clarify why regulatory RNAs and RNA protein interfaces show strong context dependence and epistasis, and why perturbations can yield non-additive effects that depend on expression level, localization, and processing (Bartel, 2018; Statello et al., 2021). For biotechnology, landscapes provide practical design rules: select for robustness across deployment relevant matrices, rather than only for peak activity in optimized buffers, and treat modular junctions and folding routes as first class design objects (Paunovska et al., 2022; Fowler and Fields, 2014; Saha et al., 2024).

### 7.4 What remains unresolved and what now looks testable

Several gaps remain central, one is continuity across steps in chemically messy environments. It is increasingly clear that isolated clean reactions are insufficient as evidence for plausibility, what matters is whether coupled sequences of steps remain compatible when side reactions and competing sinks are present, and whether environmental filters can bias mixtures toward persistent intermediates (Walton et al., 2022; Vázquez-Salazar, 2026). A second is strand dynamics and turnover. Many regimes that promote polymerization also risk duplex trapping or misfolding, so the physical processes that permit copying, separation, and reuse remain as important as monomer synthesis itself (Ianeselli et al., 2023). A third is system level selection under parasitism. Experiments on cooperative networks and sustained evolution show that population structure can stabilize function, but the mapping from compartment physics to effective error thresholds is still incomplete (Vaidya et al., 2012; Higgs and Lehman, 2015; Papastavrou et al., 2024).

These problems are not peripheral, they are direct expressions of a core genetics theme, how information-bearing polymers can produce heritable phenotypes under constraints, and how robustness and evolvability emerge from rugged landscapes and structured populations. The RNA worlds framework therefore functions as a bridge, it connects origin-of-life chemistry, cellular RNA genetics, and applied RNA engineering through shared measurable variables and shared tradeoffs.

## 8 Conclusion and outlook

### 8.1 Open questions and promising research directions

Several open problems can now be read as designable experiments. One is continuity across steps, which boundary conditions allow activated substrate generation, oligomer assembly, and copying to co-occur without mutually exclusive requirements, and with net accumulation across repeated cycles (Pressman et al., 2015; Ding et al., 2023; Muñoz-Velasco et al., 2024; Vázquez-Salazar, 2026). A second is system-level selection, how compartment type, exchange rates, and spatial structure shift parasite control and effective error thresholds, even when copying fidelity remains modest (Higgs and Lehman, 2015; Vaidya et al., 2012; Papastavrou et al., 2024). A third is catalytic breadth. Methyltransferase and alkyltransferase ribozymes already show that RNA catalysis can extend beyond classical phosphoryl transfer, so the key question is no longer whether such reactions are possible, but which additional chemistries remain accessible to compact RNAs under plausible ion compositions and cofactor regimes, and how often gains in catalytic novelty trade against increased fragility to environmental fluctuation (Scheitl et al., 2020; Scheitl et al., 2022; Okuda et al., 2023; Chen et al., 2025; Saha et al., 2024). In parallel, applied work continues to sharpen the same issues, predictive standards for folding and function across transcript contexts and cellular states remain incomplete, so robustness mapping across relevant environments remains a high-value strategy rather than an optional add-on (Fowler and Fields, 2014; Saha et al., 2024).

### 8.2 Final perspective

After 4 decades, the RNA world remains useful as a research program when it is treated as a set of quantitative constraints rather than as a single narrative. The strongest progress has come from translating plausibility debates into measurable benchmarks, yields of activated substrates, copying fidelity and processivity, strand exchange dynamics, and retention in structured populations (Pressman et al., 2015; Papastavrou et al., 2024; Vázquez-Salazar, 2026). The same strategy improves ribozyme and aptamer engineering, explicit mapping of sequence to function, careful treatment of epistasis, and selection across relevant fluctuations are often more informative than additional optimization in narrowly defined buffers (Fowler and Fields, 2014; Saha et al., 2024). On this view, ribozymes remain simultaneously mechanistic examples of what RNA catalysis can support and versatile platforms for evolution-guided design.
